# Fractional Order Total Variation Low‐Rank Representation on Single‐Cell RNA Sequencing Clustering

**DOI:** 10.1049/syb2.70070

**Published:** 2026-05-29

**Authors:** Pengcheng Yang, Fei Lu, Qianwen Xue, Weimin Ma, Qianwen Liu, Qiang Li, Yulin Zhang, Xiaochun Cheng

**Affiliations:** ^1^ College of Mathematics and Systems Science Shandong University of Science and Technology Qingdao Shandong China; ^2^ School of Information and Control Engineering Qingdao University of Technology Qingdao Shandong China; ^3^ Qingdao Key Laboratory of Multimodal Medical Big Data Modeling and Intelligent Early Warning Qingdao China; ^4^ Qingdao Maternal & Child Health and Family Planning Service Center Qingdao Shandong China; ^5^ Peking University People's Hospital Qingdao Hospital Qingdao China; ^6^ Computer Science Department, Bay Campus, Fabian Way Swansea University Swansea Wales UK

**Keywords:** biocomputing, bioinformatics, biology computing

## Abstract

Traditional bulk RNA sequencing often masks cell‐to‐cell variability, leading to a loss of individual heterogeneity information. Single‐cell RNA sequencing (scRNA‐seq) preserves cellular heterogeneity by reverse‐transcribing, amplifying, as well as sequencing mRNA molecules from individual cells, enabling in‐depth studies of cell development, differentiation, and disease mechanisms. However, scRNA‐seq data are inherently high‐dimensional and noisy with prevalent dropout events, posing challenges for accurate clustering and subtype identification. To address these issues, this study proposes an Adaptive Fractional‐Order Total Variation Regularised Low‐Rank Representation (AFTV‐LRR) model that integrates adaptive fractional‐order total variation into the low‐rank representation framework. The proposed method reconstructs low‐rank subspace structures to learn cell similarities while preserving fine‐grained cellular features through fractional‐order gradient learning. The optimisation problem is efficiently solved using the Alternating Direction Method of Multipliers (ADMM), and spectral clustering is applied to the learnt similarity matrix for accurate cell type identification. Extensive experiments on 11 publicly available scRNA‐seq datasets demonstrate that AFTV‐LRR achieves competitive and often superior performance compared with eight representative single‐cell clustering algorithms in terms of Adjusted Rand Index (ARI) and Normalised Mutual Information (NMI). Visualisation with t‐SNE further confirms that the proposed model yields clearer inter‐cluster separations and higher intra‐cluster compactness. Moreover, marker gene analysis using the mouse embryo dataset supports the biological interpretability and robustness of the clustering results. Overall, this work provides an adaptive computational framework for improving the accuracy and reliability of single‐cell clustering analysis.

## Graphical abstract

We propose an Adaptive Fractional‐Order Total Variation Regularised Low‐Rank Representation (AFTV‐LRR) model for accurate clustering of single‐cell RNA sequencing data. By integrating fractional‐order total variation into the low‐rank representation framework, our method effectively reduces noise and preserves local geometric structure. Experimental results on 11 scRNA‐seq datasets demonstrate that AFTV‐LRR outperforms six representative clustering methods in terms of ARI and NMI metrics.

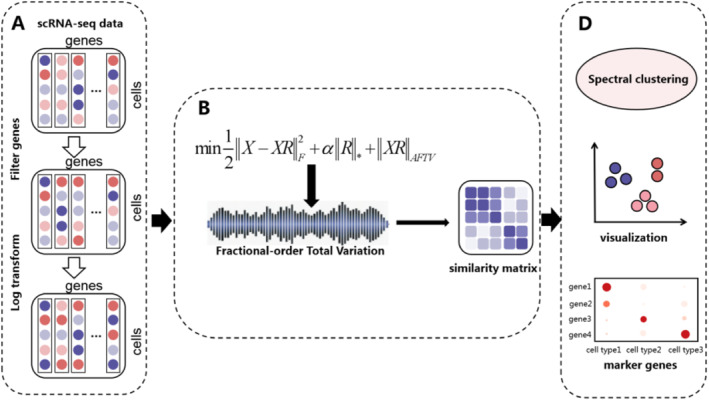

## 1 Introduction

Cell type identification is central to the studies of development, differentiation, and functional heterogeneity. Single‐cell RNA sequencing (scRNA‐seq) enables transcriptome‐wide profiling at cellular resolution, making clustering a standard step for discovering subpopulations and building cell atlases [1, 2, 3, 4, 5, 6]. Reliable clustering remains difficult because scRNA‐seq measurements are inherently noisy and biased, with strong sparsity driven by limited capture efficiency and stochastic transcription. Dropout‐dominated matrices, heterogeneous library sizes, and batch effects jointly degrade cell–cell similarity estimation and downstream partitioning.

A large body of work addresses these issues through preprocessing, representation learning, and graph‐based partitioning. Toolkits such as Seurat and Scanpy provide widely used pipelines for normalisation, feature selection, neighbour graph construction, and community‐detection‐based clustering [7]. In this setting, Louvain and its refinement Leiden are frequently adopted to partition kNN graphs at scale [8, 9]. Beyond pipeline‐level solutions, similarity learning and consensus strategies have been developed to stabilise clustering under noise and sparsity. SIMLR learns an adaptive similarity matrix via multi‐kernel learning for clustering and visualisation [10]. SC3 aggregates multiple clustering solutions into a consensus partition and provides marker discovery utilities [11]. Corr introduces a differentiability‐correlation‐based similarity and variance analysis to infer cluster structure [12].

Deep generative and deep embedding approaches offer another line of solutions by learning low‐dimensional latent variables under explicit count noise models. scVI uses variational inference to learn probabilistic embeddings for integration and downstream analysis [13]. Methods targeting denoising and signal extraction include ZINB‐WaVE and DCA, which model over‐dispersion and zero inflation to recover structured expression patterns [14, 15]. Clustering‐specific deep frameworks, such as scDeepCluster and DESC, couple representation learning with clustering objectives and can improve robustness in large datasets [16, 17].

Despite these advances, accurate clustering still depends on the quality of the cell–cell affinity graph. Many approaches emphasise pairwise similarities derived from embeddings or local neighbourhoods. Higher‐order structure and global subspace organisation can be under‐exploited, especially when noise propagates into neighbourhood graphs. Subspace and low‐rank modelling provide a complementary mechanism by separating latent structure from corruptions and enforcing global consistency. Low‐rank representation (LRR) has been used to uncover intrinsic subspace structures and to construct affinity matrices for spectral clustering. SinNLRR introduces nonnegativity into LRR for scRNA‐seq clustering [18]. SCCLRR incorporates cosine similarity to refine affinity estimation [19]. ATV‐LRR combines total variation regularisation with LRR to preserve local smoothness while suppressing noise explainability [20]. Robust extensions such as CNLLRR and CBLRR further improve resistance to outliers and kernel‐induced instability [21, 22]. Multi‐view variants also appear in the literature, including tensor‐based formulations for integrating heterogeneous measurements [23, 24].

Beyond single‐modality scRNA‐seq clustering, recent studies have extended single‐cell computational analysis to broader problem settings. For example, scMoMtF provides an interpretable multitask framework for matched single‐cell multi‐omics data analysis [25], whereas STAAAE develops an unsupervised adversarial autoencoder‐based strategy for spatial transcriptomics analysis [26]. In addition, deep learning has also been introduced into other single‐cell omics modalities, such as scMCG for single‐cell ATAC‐seq analysis [27]. From a methodological perspective, recent advances in multi‐view clustering, including multiview subspace clustering via low‐rank symmetric affinity graph and semantic‐coordinated dynamic fusion frameworks, further highlight the importance of integrating complementary structural information across views [28, 29]. Moreover, transformer‐based single‐cell language models have emerged as a promising direction for foundation‐style representation learning in single‐cell biology [30]. Although these methods address broader or different problem settings than the single‐modality scRNA‐seq clustering task considered here, they provide useful context for positioning AFTV‐LRR within the evolving landscape of modern single‐cell and clustering research.

However, within the specific context of single‐modality scRNA‐seq clustering, these broader frameworks do not directly address the challenge of learning robust cell similarities while preserving subtle cellular structures in the presence of high dropout noise. To tackle this, we propose Adaptive Fractional‐Order Total Variation Regularised Low‐Rank Representation (AFTV‐LRR). Fractional‐order regularisation provides a principled way to control smoothness while retaining fine‐scale details. In the context of scRNA‐seq data, such fine‐scale details correspond to subtle transcriptomic differences among closely related cell states, weak boundaries between transitional populations, and local expression patterns that may be obscured by dropout noise. Compared with integer‐order total variation, fractional‐order regularisation introduces a more flexible nonlocal smoothing mechanism through fractional differences, allowing information from a broader neighbourhood to contribute to regularisation. As a result, it can suppress isolated noisy fluctuations while reducing the risk of oversmoothing weak but biologically meaningful structures, such as rare subpopulations or gradual developmental transitions [31, 32]. Motivated by the need for stable affinity learning in sparse single‐cell data, the model integrates adaptive fractional‐order total variation into an LRR framework, aiming to reduce noise amplification in affinity construction while preserving local geometric features and global subspace consistency. Compared with ATV‐LRR, which relies on integer‐order total variation and mainly enforces local piecewise smoothness, AFTV‐LRR leverages fractional‐order gradients to capture multi‐scale neighbourhood interactions. This enables the learnt representation to better preserve subtle expression differences among closely related cell types, weak boundaries between transitional states, and rare subpopulations, while still suppressing isolated noise. In this way, the proposed formulation offers stronger adaptivity and improved retention of biologically meaningful local structures in similarity estimation.

The main contributions are summarised as follows:

1. We incorporate adaptive fractional‐order total variation into a low‐rank representation model for scRNA‐seq clustering, enabling joint denoising and detail‐preserving structure learning.
2. We develop an efficient ADMM‐based optimisation algorithm for AFTV‐LRR and perform spectral clustering on the learnt affinity matrix for cell type identification.
3. Experiments on 11 public scRNA‐seq datasets show that AFTV‐LRR achieves competitive and often superior clustering performance against representative baselines in terms of ARI and NMI.

The remainder of this paper is organised as follows. Section 2 presents the proposed model and optimisation procedure. Section 3 reports experimental settings and results. Section 4 concludes the paper and discusses future directions.

## 2 Methods

### 2.1 Overview of Low‐Rank Representation

In the literature, the TV function is usually chosen as the regularisation term for its capability of edge‐preserving, that is, φ(u)=TV(u). Note that [31]

(1) $$TV\left(u\right)≔\sum_{i,j=1}^{n}|\nabla u_{i,j}|=\sum_{i,j=1}^{n}\sqrt{|{\left(D_{x}^{+}u\right)}_{i,j}{|}^{2}+|{\left(D_{y}^{+}u\right)}_{i,j}{|}^{2}}$$

where

(2) $${\left(\nabla u\right)}_{i,j}=\left({\left(D_{x}^{+}u\right)}_{i,j},{\left(D_{y}^{+}u\right)}_{i,j}\right)$$

(3) $${\left(D_{x}^{+}u\right)}_{i,j}=\left\{\begin{array}{cc} u_{i+1,j}-u_{i,j}, & \text{if}\;i<n,\\ u_{1,j}-u_{n,j}, & \text{else,} \end{array}\right.$$

(4) $${\left(D_{y}^{+}u\right)}_{i,j}=\left\{\begin{array}{cc} u_{i,j+1}-u_{i,j}, & \text{if}\;j<n,\\ u_{i,1}-u_{i,n}, & \text{else.} \end{array}\right.$$

Assuming the matrix $X=\left(x_{1},x_{2},\text{⋯}\;,x_{n}\right)\in R^{m\times n}$ be the scRNA‐seq data measuring m genes in n cells. Considering that $x_{j}$ is a linear combination of bases in the dictionary matrix $a=\left(a_{1},a_{2},\text{⋯}\;,a_{n}\right)$, it can be expressed as

(5) X=AR,

where $R\in R^{n\times n}$ is the coefficient matrix corresponding to A.

Assuming that the data set X is extracted from the merged set of K sub‐spaces $S=\left(S_{1},S_{2},\text{⋯}\;,S_{K}\right)$, in order to restore the data space to their respective sub‐spaces, the raw data matrix X can encode each vector as a dictionary matrix. Then the coefficient matrix R of the low‐rank representation is expressed as

(6) $$\min_{R}\;\text{rank}\;\left(R\right),\;\text{s.t.}\;X=XR,$$

the formula is further changed as follows:

(7) $$\min_{R,E}\;\text{rank}\;\left(R\right)+\alpha \|E\|,\;\text{s.t.}\;X=XR+E,$$

where $R\in R^{n\times n}$ represents the coefficient matrix and rank(R) represents the rank of the matrix. $E\in R^{m\times n}$ is the noise term with different norm constraints, such as $L_{1}$ norm and $L_{0}$ norm, which can characterise the sparsity of the error term E when $L_{1}$ norm is used.

Formula (7) is usually difficult to solve directly because the kernel norm of matrix is the matrix rank envelope. In order to be able to formulate (7) with convex relaxation, the matrix kernel norm is the sum of the singular values in the matrix. R is replaced with V to relax the constraints. In contrast, if the expression of cells is described from the same subspace, these cells are more likely to belong to the same type, which requires non‐negative constraints on the coefficient matrix, keeping the middle element equal to or greater than zero to reflect the non‐negative similarity of cells of the same type. So Equation (7) can be expressed as follows:

(8) $$\min_{R}\frac{1}{2}\|X-XR{\|}_{F}^{2}+\alpha \|R{\|}_{*},\;\text{s.t.}\;R\geq 0.$$

On the one hand, the low‐rank representation method can suppress the noise well to a certain extent. On the other hand, it can better obtain the subspace structure embedded in the data, characterise the global features of the data, and explore the lowest rank representation of the data that can reflect the data among all features.

### 2.2 AFTV Regularisation Term

In the process of clustering of scRNA data, the boundary division of clusters, retaining the original information and the original structure will have a very important impact on the results of the clustering. The richer the information of the retained data, the accuracy of the clustering will also improve. From the perspective of mathematics, in order to enhance the denoised effect of the model and restore the original information of the data, the relevant mathematical methods must be used, so that the model can not only remove the noise but also retain the boundaries and details of the data as much as possible. Therefore, we consider using an adaptive fractional total variation regularity term to constrain the model, an energy functional *E* is used to represent the adaptive fractional total variation regular term. Let $\Omega \subset R^{2}$ be the spatial domain and H:Ω→R denote the underlying signal (in our setting, H represents the data arranged on a 2D grid for defining fractional‐order operators). We define the AFTV energy functional as

(9) $$E\left(H\right)=\|H{\|}_{\mathrm{AFTV}}.$$

Following the definition of fractional‐order total variation, for an order β>0, we define

(10) $$E\left(H\right)=\sup\left\{\int_{\Omega }H\;{div}^{\beta }\Phi \;dx\text{:}\Phi =\left(\phi_{1},\phi_{2}\right)\in {\left(C_{0}^{\beta }\left(\Omega \right)\right)}^{2},\;|\phi_{i}{|}_{L_{\infty }\left(\Omega \right)}\leq 1,\;i=1,2\right\},$$

where $C_{0}^{\beta }\left(\Omega \right)$ denotes the space of β‐order continuously differentiable functions with compact support in Ω, and Φ is a vector‐valued test function. The fractional‐order divergence is defined by

(11) $${div}^{\beta }\Phi =\frac{\partial^{\beta }\phi_{1}}{\partial x^{\beta }}+\frac{\partial^{\beta }\phi_{2}}{\partial y^{\beta }},$$

where $\frac{\partial^{\beta }\phi_{i}}{\partial z^{\beta }}$ denotes the β‐order derivative along direction z∈{x,y}(i=1,2). In this work, we adopt the Grünwald–Letnikov (GL) fractional derivative for convenient discretisation and computation [31, 32, 33].

We define the space of functions with bounded β‐order total variation as

(12) $$BV^{\beta }\left(\Omega \right)≔\left\{\;H\in L^{1}\left(\Omega \right)\text{:}TV^{\beta }\left(H\right)<\infty \;\right\}.$$

For $H\in BV^{\beta }\left(\Omega \right)$, we discretise Ω on a rectangular grid $\left\{\left(x_{i},y_{j}\right)\text{:}1\leq i\leq N,\;1\leq j\leq M\right\}$, and represent the discretised data as a matrix $H\in R^{N\times M}$ with entries $H_{i,j}=H\left(x_{i},y_{j}\right)$. Based on the GL fractional derivative, we define the discrete fractional‐order derivatives along the x‐ and y‐directions (equivalently, the discrete fractional‐order gradient $\nabla^{\beta }H≔\left(D_{1}^{\beta }H,D_{2}^{\beta }H\right)$) by

(13) $$\left\{\begin{array}{c} {\left(D_{1}^{\beta }H\right)}_{i,j}=\sum_{k=0}^{K-1}{\left(-1\right)}^{k}C_{k}^{\beta }H_{i-k,j},\\ {\left(D_{2}^{\beta }H\right)}_{i,j}=\sum_{k=0}^{K-1}{\left(-1\right)}^{k}C_{k}^{\beta }H_{i,j-k}, \end{array}\right.$$

where K is the truncation length (i.e., the number of adjacent points used to approximate the fractional derivative).

The advantage of this fractional‐order formulation is that the discrete operators $D_{1}^{\beta }$ and $D_{2}^{\beta }$ aggregate weighted differences over multiple neighbouring positions rather than relying solely on first‐order local differences. This allows the regularisation to consider a broader neighbourhood, making it less sensitive to isolated dropout‐induced noise while preserving coherent local variations. For scRNA‐seq clustering, such coherent variations correspond to subtle expression differences among closely related cell types, weak boundaries between transitional states, and rare subpopulations. In other words, fractional‐order regularisation helps to suppress spurious fluctuations caused by technical noise while retaining fine‐grained biological features that are important for accurate affinity estimation and subsequent clustering.

The coefficients ${\left\{C_{k}^{\beta }\right\}}_{k=0}^{K-1}$ are given by

(14) $$C_{k}^{\beta }=\frac{\Gamma \left(\beta +1\right)}{\Gamma \left(k+1\right)\Gamma \left(\beta +1-k\right)},$$

and Γ(⋅) is the Gamma function.

The discrete fractional total variation for H is defined as [23]

(15) $$J\left(H\right)=\|\nabla^{\beta }H{\|}_{1}≔\sum_{i,j}\left(\left|{\left(D_{1}^{\beta }H\right)}_{i,j}|+|{\left(D_{2}^{\beta }H\right)}_{i,j}\right|\right),$$

and the corresponding discrete fractional divergence operator is given by

(16) $${\left({div}^{\beta }p\right)}_{i,j}={\left(-1\right)}^{\beta }\sum_{k=0}^{K-1}{\left(-1\right)}^{k}C_{k}^{\beta }\left(p_{i+k,j}^{\left(1\right)}+p_{i,j+k}^{\left(2\right)}\right),$$

where $p^{\left(1\right)}$ and $p^{\left(2\right)}$ denote the two components of the vector field p.

To introduce adaptivity, we employ a spatially varying exponent l(x,y) that controls the strength of diffusion in different regions, satisfying

(17) 1<l(x,y)<2.

l(x,y) is determined from local gradient information so that relatively homogeneous regions are smoothed more strongly, whereas regions with sharp transitions are smoothed more conservatively. With this adaptive mechanism, the gradient of the fractional regularisation term E(H) is

(18) $$\nabla E=D_{1}^{\beta *}\left(\frac{D_{1}^{\beta }H}{|D^{\beta }H{|}^{2-l\left(x,y\right)}}\right)+D_{2}^{\beta *}\left(\frac{D_{2}^{\beta }H}{|D^{\beta }H{|}^{2-l\left(x,y\right)}}\right),$$

where $D_{1}^{\beta *}$ and $D_{2}^{\beta *}$ denote the adjoint operators of $D_{1}^{\beta }$ and $D_{2}^{\beta }$, respectively, and $\left|D^{\beta }H\right|$ denotes the magnitude associated with the fractional‐order gradient. Since ${\left(\nabla^{\beta }\right)}^{*}={\left(-1\right)}^{\beta }{div}^{\beta }$, the corresponding gradient‐descent flow can be expressed as

(19) $$\frac{\partial H}{\partial t}=-\left[D_{x}^{\beta *}\;\left(\frac{D_{x}^{\beta }H}{|D^{\beta }H{|}^{2-l\left(x,y\right)}}\right)+D_{y}^{\beta *}\;\left(\frac{D_{y}^{\beta }H}{|D^{\beta }H{|}^{2-l\left(x,y\right)}}\right)\right].$$

### 2.3 AFTV‐LRR Model

**FIGURE 1:**
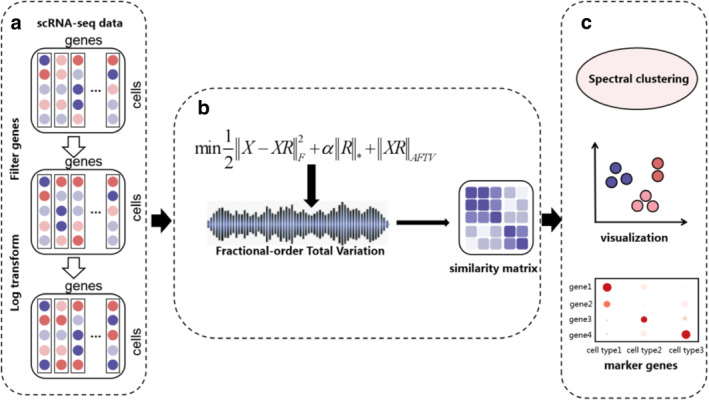
The overall framework of the proposed adaptive fractional total variation low‐rank representation (AFTV‐LRR) model. The process includes gene filtering, normalisation, similarity learning via AFTV‐LRR, and spectral clustering based on the learnt similarity matrix. (a) Preprocessing of scRNA‐seq data, including gene filtering and log transformation, (b) Construction of the AFTV‐LRR model with fractional‐order total variation to obtain the similarity matrix, (c) Spectral clustering and downstream analysis, including visualization and marker gene identification.

Based on the above formulations, we integrate the adaptive fractional‐order total variation term into the low‐rank representation framework to jointly achieve noise suppression and structure preservation. The overall framework of the proposed AFTV‐LRR model is shown in Figure 1. The model aims to achieve robust similarity learning for scRNA‐seq data by introducing the adaptive fractional total variation (AFTV) regularisation into the LRR framework.

Traditional low‐rank representation (LRR) models have been widely used to capture global subspace structures in high‐dimensional single‐cell expression data. However, scRNA‐seq measurements are frequently affected by technical noise and dropout, which may distort local neighbourhood geometry and degrade similarity learning if only global low‐rankness is enforced. To better preserve local structures while suppressing noise, we incorporate the proposed AFTV regularisation on the reconstructed representation XR, leading to the AFTV‐regularised low‐rank model. Compared with ATV‐LRR, which relies on integer‐order total variation and mainly enforces local piecewise smoothness, AFTV‐LRR leverages fractional‐order gradients to capture multi‐scale neighbourhood interactions. This enables the learnt representation to better preserve subtle expression differences among closely related cell types, weak boundaries between transitional states, and rare subpopulations, while still suppressing isolated noise. In this way, the adaptive fractional‐order mechanism helps maintain biologically meaningful local structures that may be lost under integer‐order regularisation.

Before modelling, we perform a gene filtering step by removing the lowest 5% of genes whose expression values are mostly zeros. Then, $L_{2}$‐normalisation is applied to eliminate scale differences between samples, ensuring comparability across cells.

Let $X\in R^{m\times n}$ denote the gene expression matrix with m genes and n cells. We learn a coefficient matrix $R\in R^{n\times n}$ and formulate AFTV‐LRR as

(20) $$\min\frac{1}{2}\|X-XR{\|}_{F}^{2}+\alpha \|R{\|}_{*}+\|XR{\|}_{\mathrm{AFTV}},\;s.t.\;R\geq 0,$$

where $\|\cdot {\|}_{F}$ is the Frobenius norm, $\|\cdot {\|}_{*}$ is the nuclear norm promoting a low‐rank structure, α>0 controls the low‐rankness strength, and $\|\cdot {\|}_{\mathrm{AFTV}}$ denotes the adaptive fractional total variation regularisation defined in the previous subsection (including the adaptive mechanism governed by l(x,y)∈(1,2) and the fractional order β).

We adopt the squared Frobenius norm $\frac{1}{2}\|X-XR{\|}_{F}^{2}$ as the data‐fidelity term. This choice is standard in LRR‐type formulations and corresponds to an i.i.d. Gaussian noise assumption. More importantly, it yields a smooth and convex fidelity that enables efficient and numerically stable optimisation under ADMM, which is consistent with our lightweight design goal. We note that alternative robust fidelities (e.g., $\ell_{1}$ or $\ell_{2,1}$ norms) may further enhance robustness under heavy‐tailed noise or outliers but typically lead to more complex and computationally heavier subproblems; exploring such variants is left for future work.

To solve the constrained and non‐smooth optimisation problem, we adopt the Alternating Direction Method of Multipliers (ADMM). We introduce an auxiliary variable $Q\in R^{n\times n}$ to decouple the nuclear‐norm term and the AFTV regulariser, yielding the equivalent problem:

(21) $$\min\frac{1}{2}\|X-XR{\|}_{F}^{2}+\alpha \|Q{\|}_{*}+\|XR{\|}_{\mathrm{AFTV}},\;\text{s.t.}\;R\geq 0,\;Q-R=0.$$

The corresponding augmented Lagrangian function is defined as

(22) $$L\left(R,Q,Y\right)=\frac{1}{2}\|X-XR{\|}_{F}^{2}+\alpha \|Q{\|}_{*}+\|XR{\|}_{\mathrm{AFTV}}+Y^{T}\left(Q-R\right)+\frac{1}{2\mu }\|Q-R{\|}_{F}^{2},$$

where Y is the Lagrange multiplier and μ>0 is a penalty parameter. ADMM alternates among R, Q, and Y using

(23) $$\left\{\begin{array}{c} R^{l+1}=\arg\min_{R}\;L\left(R,Q^{l},Y^{l}\right),\\ Q^{l+1}=\arg\min_{Q}\;L\left(R^{l+1},Q,Y^{l}\right),\\ Y^{l+1}=Y^{l}+\frac{1}{\mu }\left(R^{l+1}-Q^{l+1}\right). \end{array}\right.$$

Step 1 Sub‐problem of R.

To update R, we fix Q and Y and keep only the terms related to R. The R‐subproblem is

(24) $$R^{l+1}=\arg\;\min_{R}\;L\left(R,Q^{l},Y^{l}\right)$$

and the iterative form of R is obtained by taking the derivative and setting it to zero:

(25) $$R^{l+1}=\frac{X^{T}X+Y^{l}+\frac{1}{\mu }Q^{l}+X^{T}\text{div}\;\;\left[\nabla \;\left(\frac{\nabla_{R_{x}}}{\nabla^{\beta }R}\right)+\nabla \;\left(\frac{\nabla_{R_{y}}}{\nabla^{\alpha }R}\right)\right]}{X^{T}X+\frac{1}{\mu }I}.$$

In practice, the non‐negativity constraint is enforced by projecting R onto the nonnegative orthant after each update, that is, R←max(R,0).

Step 2 Sub‐problem of Q.

Fixing R and Y, the Q‐subproblem is solved by singular value thresholding (SVT) [34]. First, we keep only the terms related to Q:

(26) $$Q^{l+1}=\arg\min_{Q}\;L\left(R^{l+1},Q,Y^{l}\right)$$

Then it is solved by the thresholding operator:

(27) $$Q^{l+1}={\text{Soft}\;}_{\mu ,Q}\;\left(R^{l+1}-\mu Y^{l}\right),$$

where Soft(⋅) denotes the SVT operator applied to the singular values.

Step 3 Sub‐problem of Y.

Fixing the other variables, the multiplier is updated as

(28) $$Y^{l+1}=Y^{l}+\frac{1}{\mu }\left(R^{l+1}-Q^{l+1}\right),$$

with initialisation $Y^{0}=0$.

After all variables are updated, we symmetrise the learnt coefficient matrix to obtain the final similarity matrix:

(29) $$S=\frac{\left(R+R^{T}\right)}{2}.$$

The ADMM procedure is summarised in Table 1. After obtaining the similarity matrix S, spectral clustering is applied to derive the final clustering results. This process enables the identification of distinct and biologically meaningful cell populations, and the clustering performance will be further illustrated and analysed in the subsequent sections.

**TABLE 1:** AFTV‐LRR algorithm flow under ADMM.

Algorithm: AFTV‐LRR
**Input:** $X\in R^{m\times n}$; parameters α,μ; Maxiter; tolerance ε
**Initialise:** $R^{0}=Q^{0}=Y^{0}=0$; l=0
**Repeat:**
Update R by Formula (24) and project R←max(R,0);
Update Q by Formula (26) and project Q←max(Q,0);
Update Y by Formula (28);
l=l+1
**Until:** $\|R^{l+1}-R^{l}{\|}_{F}<\varepsilon$ **or** l= Maxiter
**Output:** R

### 2.4 Convergence Analysis

The proposed AFTV‐LRR model involves three variables, namely R, Q, and Y, and the resulting optimisation problem is non‐smooth and non‐convex due to the presence of the nuclear norm and the adaptive fractional total variation regularisation. Therefore, providing a strict global convergence guarantee is generally challenging. In this work, we adopt the alternating direction method of multipliers (ADMM) to solve the proposed model, and we assess convergence from both theoretical and empirical perspectives.

ADMM has well‐established convergence properties for convex problems under mild conditions [35]. Although the overall objective of AFTV‐LRR is non‐convex, each ADMM update in our solver corresponds to minimising an augmented Lagrangian subproblem with respect to one variable while fixing the others. In particular, the Q‐subproblem with nuclear‐norm regularisation is solved via the singular value thresholding (SVT) operator [34], which yields the optimal solution of the proximal subproblem. The remaining updates are computed efficiently within the ADMM framework, whereas the Lagrange multiplier is updated in a standard manner. Here, *μ* denotes the penalty parameter of the augmented Lagrangian, which controls the strength of the quadratic penalty term enforcing the equality constraints; a larger *μ* typically enforces constraints more strongly but may require careful tuning to balance primal and dual progress.

**FIGURE 2:**
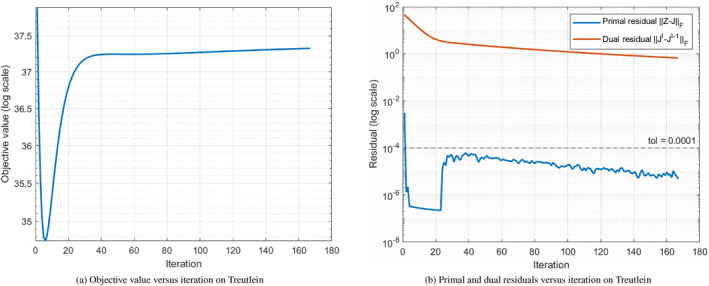
Convergence behaviour of the ADMM solver for AFTV‐LRR on the Treutlein dataset.

To further substantiate convergence in practice, we explicitly monitor the objective value and the ADMM residuals across iterations. Figure 2 reports a representative example on the Treutlein dataset. As shown in Figure 2a, the objective value quickly approaches a stable level after a number of iterations and then remains nearly unchanged. Meanwhile, Figure 2b shows that the primal residual and dual residual decrease and remain small throughout the iterations, indicating stable convergence behaviour of the proposed solver. Similar convergence trends were observed on other datasets; due to space limitations we report one representative example.

## 3 Results

### 3.1 Evaluating Indicators

To assess the performance of the clustering methods, we chose four popular clustering evaluation metrics: Normalised Mutual Information (NMI) and Adjusted Rand Index (ARI). NMI is calculated as

(30) $$NMI=\frac{2I\left(X\text{;}Y\right)}{H\left(X\right)+H\left(Y\right)}$$

where I(X;Y) represents the mutual information between X and Y, and H is the entropy function.

The range of values for NMI is [0, 1], where larger values indicate better clustering. The formula of the Rand coefficient is

(31) $$RI=\frac{a+b}{C_{2}^{n}}$$

where a is the number of pairs correctly labelled in the same set and b is the number of pairs correctly labelled as not in the same data set.

The formula for the ARI can be expressed as follows:

(32) $$ARI=\frac{RI-E\left(RI\right)}{\max\left(RI\right)-E\left(RI\right)}.$$

ARI ranges from [−1,1], and larger values indicate better clustering. E(RI) is the expected RI of random labelling.

### 3.2 Datasets and Preprocessing

We collect 12 publicly available scRNA‐seq datasets to test and validate the performance of the clustering method, where the cell types are known in advance or validated in the respective studies. These datasets have been widely used as benchmark datasets in recent single‐cell clustering studies. The number of cells ranges from 80 to 2903, and the number of clusters ranges from 3 to 11. Download links for these scRNA‐seq datasets can be found at https://github.com/ZzzOctopus/scASGC. Detailed information is given in Table 2.

**TABLE 2:** Details of the scRNA‐seq datasets used in this study.

Datasets	Cells	Genes	Clusters	Platforms
Treutlein [36]	80	959	5	Illumina‐seq
Ting [37]	114	14,405	5	Illumina‐seq
Yan [38]	124	3840	8	Illumina‐seq
Pollen [39]	249	14,805	10	Smart‐seq
Ginhoux [40]	251	11,834	3	Smart‐seq
Darmanis [41]	420	22,085	8	Smart‐seq
Zheng [4]	500	4776	3	Smart‐seq
Usoskin [42]	622	17,772	4	Smart‐seq
Kolod [43]	704	10,685	3	Smart‐seq
Klein [44]	2717	24,175	4	inDrop
Zeisel [45]	2903	14,890	7	STRT‐seq
Mouse embryo (Deng) [46]	268	22,431	6	Smart‐seq

*Note:* The Mouse embryo (Deng) dataset is used only for marker gene identification and biological interpretation, and is not included in the clustering benchmark comparisons (Tables 3 and 4).

Preprocessing is divided into three steps: gene filtering, data log‐transformation and normalisation. A gene will be removed if it contains more than 95% of zero elements in all cells. $x_{ij}$ of the matrix X is log‐transformed after adding the pseudo‐count of 1, that is,

(33) $$x_{ij}=\log_{2}\left(x_{ij}+1\right).$$

The data is then normalised by

(34) $$x_{ij}=\log_{2}\left(\frac{x_{ij}}{\sum_{j=1}^{n}x_{ij}}\times 10^{5}+1\right).$$

### 3.3 Parameter Selection

AFTV‐LRR involves two key hyperparameters that are specified manually: the fractional order β in the fractional‐order total variation regulariser and the neighbourhood size K, which determines the number of adjacent points used to compute the discrete fractional derivative. In essence, β controls the strength of fractional‐order smoothing, whereas K regulates the amount of local neighbourhood information incorporated into the model. Appropriate parameter choices are therefore important for achieving stable and accurate clustering results.

**FIGURE 3:**
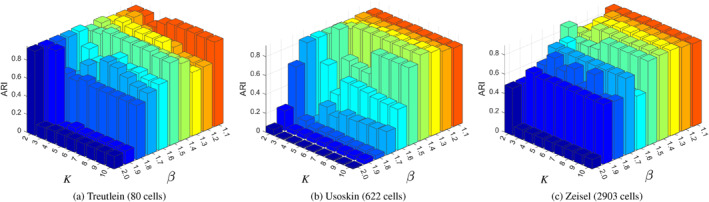
Sensitivity analysis of AFTV‐LRR parameters β and K under the ARI metric on datasets of different sizes.

To assess the robustness of these parameters across different dataset scales, we conduct a sensitivity analysis on three representative scRNA‐seq datasets with increasing numbers of cells: Treutlein (80 cells), Usoskin (622 cells), and Zeisel (2903 cells). For each dataset, we evaluate the clustering performance measured by ARI under different combinations of (β,K), and the results are summarised in Figure 3.

As shown in Figure 3, AFTV‐LRR generally exhibits a stable high‐performance region when β is relatively small (approximately 1.1–1.5). When β becomes too large (e.g., β≥1.7), the imposed regularisation may lead to oversmoothing, which can blur intrinsic cluster boundaries and consequently reduce ARI. With respect to K, the optimal choice is more dataset‐dependent. For the large‐scale Zeisel dataset, an excessively small neighbourhood (e.g., K=2) is typically insufficient to capture local manifold structures, whereas K≥3 yields substantially more stable performance. For the smaller Treutlein dataset and the medium‐scale Usoskin dataset, the model is comparatively less sensitive to K once β is within the preferred range, forming a broad plateau of strong performance across moderate K values.

In practice, we select (β,K) for each dataset by choosing the combination that maximises ARI within the robust regions identified above, and we use the resulting optimal parameters in subsequent experiments.

### 3.4 Results

#### 3.4.1 Comparison With Other Clustering Methods

To validate the clustering performance of the proposed AFTV‐LRR, we conducted experiments on 11 publicly available scRNA‐seq datasets and compared it with eight representative baselines, including ATV‐LRR [20], SIMLR [10], SinNLRR [18], SC [47], SSC [48], MPSSC [49], JLONMFSC [50], and DSINMF [51]. Among them, JLONMFSC and DSINMF are recent matrix factorisation/deep factorization‐based clustering methods for scRNA‐seq data, which broaden the comparison across different methodological categories. For SIMLR, the number of nearest neighbours was set to 30 when the number of cells exceeded 250 and to 10 otherwise; other parameters followed the default settings recommended in the original implementation. All remaining baselines were executed with their default settings.

To account for randomness (e.g., initialisation), we repeated each method for 30 runs with different random seeds and report the results as mean ± standard deviation. For a fair optimisation budget, the maximum number of iterations was set to 100 for all iterative methods. Clustering performance was evaluated using ARI and NMI, and the results are summarised in Tables 3 and 4.

**TABLE 3:** Comparison of clustering performance (ARI metric) on 11 scRNA‐seq datasets.

Datasets	AFTV‐LRR	ATV‐LRR	SIMLR	SinNLRR	SC	SSC	MPSSC	JLONMFSC	DSINMF
Treutlein	**0.9587** ± **0.0050**	0.8741 ± 0.0052	0.5154 ± 0.0468	0.6464 ± 0.0219	0.5269 ± 0.0367	0.5185 ± 0.0298	0.6299 ± 0.0156	0.6958 ± 0.0062	0.5333 ± 0.0060
Ting	0.9161 ± 0.0144	**0.9166** ± **0.0121**	0.6683 ± 0.0402	0.5109 ± 0.0832	0.5397 ± 0.0174	0.5471 ± 0.0373	0.7841 ± 0.0127	0.8651 ± 0.0078	0.8529 ± 0.0079
Yan	**0.7652** ± **0.0054**	0.7624 ± 0.0080	0.6526 ± 0.0274	0.5620 ± 0.0200	0.3344 ± 0.0289	0.3698 ± 0.0332	0.6322 ± 0.0116	0.7424 ± 0.0079	0.7126 ± 0.0065
Pollen	0.9419 ± 0.0033	**0.9693** ± **0.0012**	0.9545 ± 0.0047	0.8542 ± 0.0202	0.6345 ± 0.0748	0.6472 ± 0.0416	0.8902 ± 0.0131	0.9365 ± 0.0026	0.9224 ± 0.0035
Ginhoux	**0.4910** ± **0.0113**	0.4709 ± 0.0130	0.4150 ± 0.0455	0.4123 ± 0.0170	0.1236 ± 0.0369	0.1607 ± 0.0141	0.3615 ± 0.0793	0.3161 ± 0.0266	0.2628 ± 0.0322
Darmanis	0.8731 ± 0.0052	0.8114 ± 0.0185	0.2991 ± 0.0671	0.6091 ± 0.0436	0.4445 ± 0.0609	0.4441 ± 0.0337	0.6153 ± 0.0568	**0.8925 ** ± **0.0052**	0.8491 ± 0.0113
Zheng	**0.9941** ± **0.0019**	**0.9941** ± **0.0021**	0.8314 ± 0.0226	0.7812 ± 0.0108	0.6712 ± 0.0496	0.6446 ± 0.0342	0.8721 ± 0.0292	0.9826 ± 0.0109	0.9695 ± 0.0022
Usoskin	**0.9016** ± **0.0026**	0.8908 ± 0.0032	0.7788 ± 0.0321	0.8727 ± 0.0159	0.6243 ± 0.0652	0.5470 ± 0.0752	0.6243 ± 0.0335	0.8766 ± 0.0092	0.8486 ± 0.0154
Kolod	**0.8290** ± **0.0132**	0.8169 ± 0.0135	0.7425 ± 0.0232	0.7971 ± 0.0108	0.4784 ± 0.0904	0.5090 ± 0.0687	0.6914 ± 0.0413	0.7424 ± 0.0081	0.7262 ± 0.0196
Klein	**0.9516** ± **0.0067**	0.9372 ± 0.0067	0.8832 ± 0.0312	0.8898 ± 0.0108	0.6268 ± 0.0614	0.6011 ± 0.0377	0.8354 ± 0.0282	0.9281 ± 0.0166	0.7174 ± 0.0316
Zeisel	**0.8492** ± **0.0155**	0.8002 ± 0.0103	0.7742 ± 0.0312	0.7882 ± 0.0108	0.5628 ± 0.0614	0.5534 ± 0.0377	0.7129 ± 0.0377	0.7584 ± 0.0094	0.6114 ± 0.0247

*Note:* Results are reported as mean ± standard deviation over 30 runs. The best performance in each dataset is highlighted in bold. When two methods achieve the same best result, both are highlighted.

**TABLE 4:** Comparison of clustering performance (NMI metric) on 11 scRNA‐seq datasets.

Datasets	AFTV‐LRR	ATV‐LRR	SIMLR	SinNLRR	SC	SSC	MPSSC	JLONMFSC	DSINMF
Treutlein	**0.8982** ± **0.0247**	0.8274 ± 0.0279	0.6874 ± 0.0632	0.6561 ± 0.0454	0.8168 ± 0.0223	0.7060 ± 0.0490	0.5383 ± 0.0900	0.7203 ± 0.0082	0.6947 ± 0.0058
Ting	**0.9197** ± **0.0044**	**0.9197** ± **0.0061**	0.6278 ± 0.0471	0.8708 ± 0.0162	0.6549 ± 0.0431	0.5323 ± 0.1016	0.7683 ± 0.0311	0.8942 ± 0.0102	0.8893 ± 0.0083
Yan	**0.8236** ± **0.0044**	0.8144 ± 0.0123	0.7272 ± 0.0262	0.6745 ± 0.0526	0.6893 ± 0.0120	0.7211 ± 0.0231	0.6903 ± 0.0105	0.8013 ± 0.0083	0.7631 ± 0.0092
Pollen	0.9433 ± 0.0040	**0.9674** ± **0.0019**	0.9034 ± 0.0152	0.8564 ± 0.0254	0.6658 ± 0.0657	0.6439 ± 0.0528	0.8078 ± 0.0328	0.9512 ± 0.0064	0.9313 ± 0.0122
Ginhoux	**0.4730** ± **0.0073**	0.4366 ± 0.0126	0.3150 ± 0.0415	0.4296 ± 0.0242	0.2156 ± 0.0402	0.2535 ± 0.0193	0.4179 ± 0.0321	0.3528 ± 0.0145	0.2683 ± 0.0368
Darmanis	0.8470 ± 0.0092	0.8104 ± 0.0120	0.6345 ± 0.0385	0.7569 ± 0.0436	0.6360 ± 0.0337	0.6278 ± 0.0236	0.6264 ± 0.0641	**0.8773** ± **0.0086**	0.8212 ± 0.0098
Zheng	0.9281 ± 0.0103	**0.9856** ± **0.0020**	0.7845 ± 0.0378	0.9689 ± 0.0028	0.4599 ± 0.0458	0.4582 ± 0.0508	0.4885 ± 0.0529	0.9727 ± 0.0095	0.9557 ± 0.0014
Usoskin	0.8727 ± 0.0159	**0.9222** ± **0.0129**	0.8320 ± 0.0378	0.8727 ± 0.0072	0.5756 ± 0.0614	0.6801 ± 0.0592	0.7823 ± 0.0378	0.8913 ± 0.0138	0.8778 ± 0.0084
Kolod	**0.8315** ± **0.0103**	0.7812 ± 0.0129	0.7490 ± 0.0312	0.8233 ± 0.0071	0.5021 ± 0.0614	0.5277 ± 0.0450	0.7011 ± 0.0450	0.7849 ± 0.0076	0.771 ± 0.0095
Klein	**0.9203** ± **0.0103**	0.9036 ± 0.0103	0.8702 ± 0.0312	0.8414 ± 0.0108	0.6397 ± 0.0614	0.6021 ± 0.0377	0.8230 ± 0.0377	0.8853 ± 0.0153	0.7614 ± 0.0294
Zeisel	**0.8211** ± **0.0103**	0.7700 ± 0.0129	0.7481 ± 0.0312	0.7512 ± 0.0108	0.5336 ± 0.0614	0.5334 ± 0.0377	0.6923 ± 0.0450	0.7734 ± 0.0203	0.6121 ± 0.0187

*Note:* Results are reported as mean ± standard deviation over 30 runs. The best performance in each dataset is highlighted in bold. When two methods achieve the same best result, both are highlighted.

From Tables 3 and 4, several observations can be made:

1. Regarding the Adjusted Rand Index (ARI), AFTV‐LRR achieves the best performance on seven datasets and shares the top result with ATV‐LRR on the Zheng dataset. Notably, JLONMFSC outperforms AFTV‐LRR on the Darmanis dataset.
2. For the Normalized Mutual Information (NMI), AFTV‐LRR obtains the best performance on six datasets. ATV‐LRR outperforms AFTV‐LRR on three datasets, namely Pollen, Zheng, and Usoskin, while JLONMFSC achieves a higher NMI than AFTV‐LRR on the Darmanis dataset. AFTV‐LRR and ATV‐LRR achieve identical NMI scores on the Ting dataset.
3. Overall, AFTV‐LRR demonstrates competitive mean performance with small variances across diverse scRNA‐seq datasets, indicating its robustness and adaptability to varying data characteristics. Compared with similarity‐based methods, such as SIMLR, and methods that do not explicitly learn tailored representations for scRNA‐seq‐specific noise, such as SC and SSC, the proposed framework integrates low‐rank representation learning with adaptive fractional‐order regularisation. This design enhances robustness to dropout noise and preserves intrinsic cellular structures, thereby facilitating accurate and reliable clustering.

**FIGURE 4:**
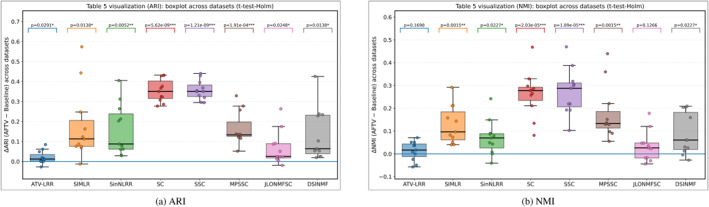
Paired one‐sided *t*‐tests (Holm‐corrected) on dataset‐wise performance differences between AFTV‐LRR and each baseline.

To quantify whether the observed performance gains are statistically significant, we performed paired one‐sided *t*‐tests across datasets. For each baseline method, we computed dataset‐wise paired differences ΔARI and ΔNMI, and tested the alternative hypothesis Δ>0. To control the family‐wise error rate under multiple comparisons, Holm correction was applied to the resulting *p*‐values. Figure 4 presents the distributions of ΔARI and ΔNMI across datasets, with Holm‐adjusted *p*‐values annotated for each baseline.

To further inspect clustering structures, we used t‐SNE [52, 53] for two‐dimensional visualisation. AFTV‐LRR first learns a low‐rank representation and constructs a cell–cell similarity matrix using the Pearson correlation. The similarity matrix is then embedded into two dimensions using t‐SNE. We compared the visualisation results of AFTV‐LRR with ATV‐LRR and SinNLRR, as shown in Figure 5.

FIGURE 5Visualisation results on 11 scRNA‐seq datasets. From this figure, it can be observed that AFTV‐LRR produces tighter intra‐cluster groupings and clearer inter‐cluster separations compared with ATV‐LRR and SinNLRR, indicating that the proposed model better preserves intrinsic manifold structures and mitigates dropout noise effects.

**Figure:**
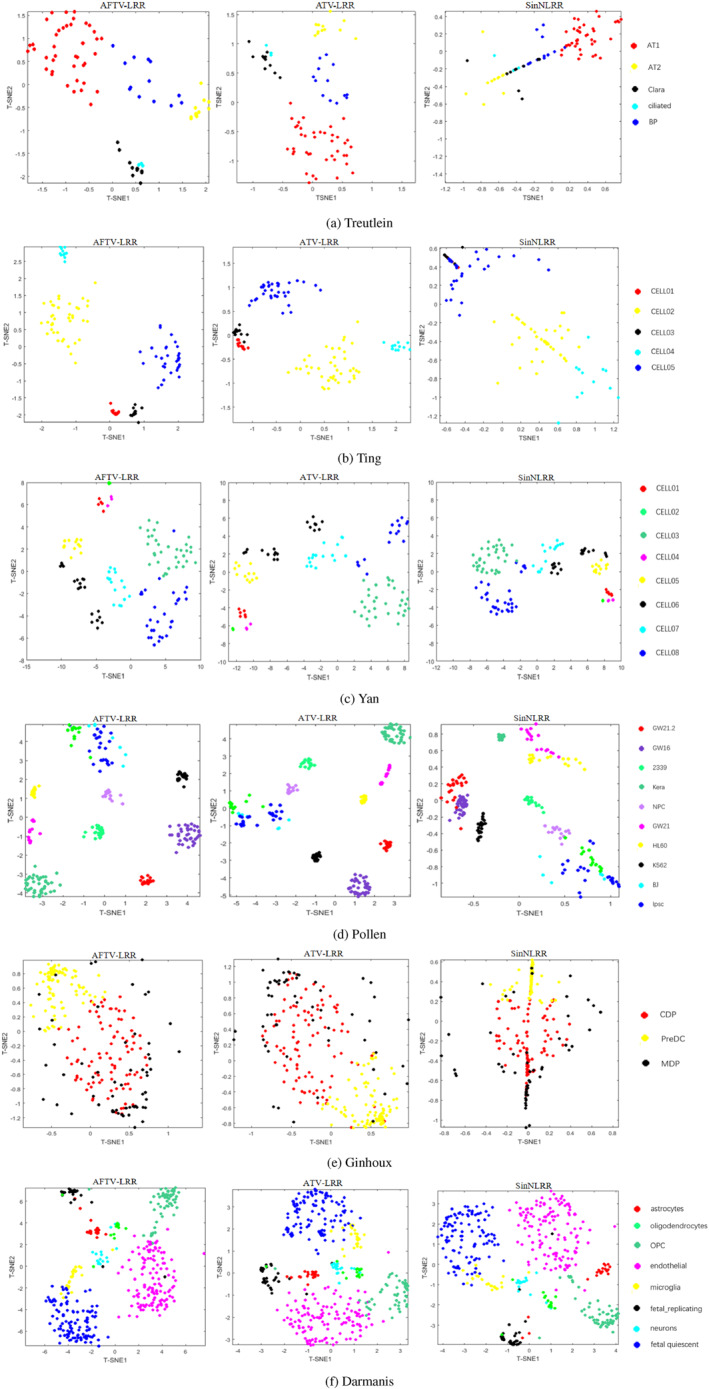

**Figure:**
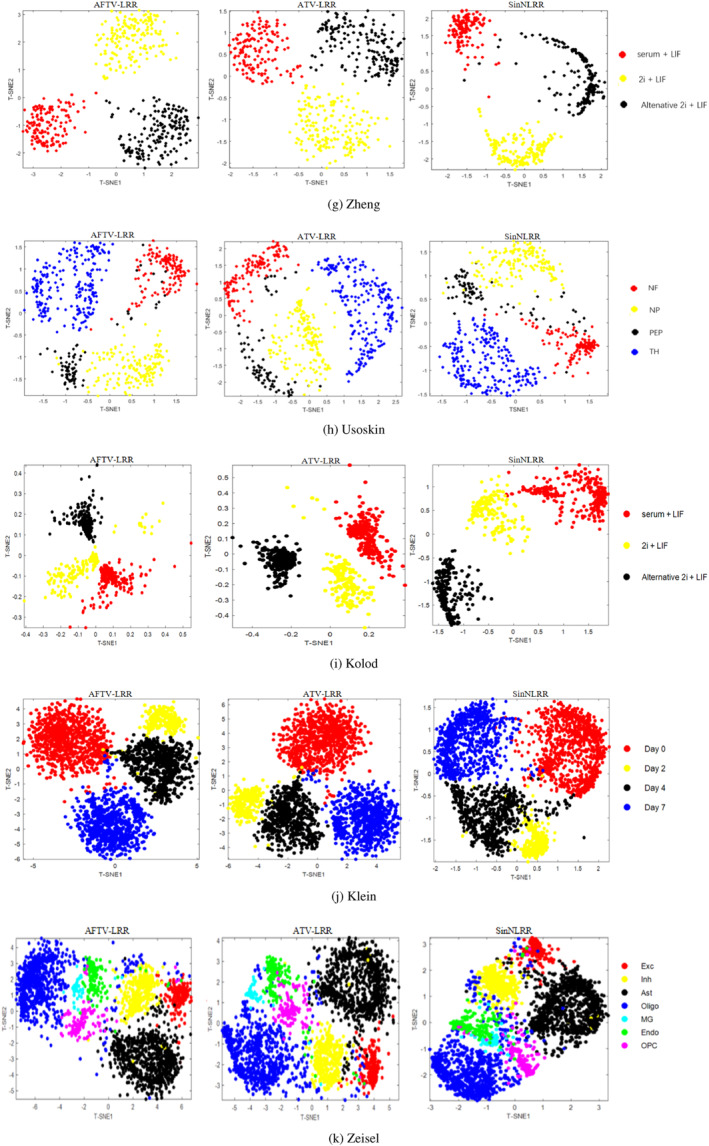

Figure 5 shows the visualisation results on 11 single‐cell datasets. Overall, the proposed AFTV‐LRR yields more compact cell distributions within clusters and clearer separation among different clusters. For example, on the Darmanis dataset, AFTV‐LRR produces well‐separated groups with reduced overlap between distinct cell populations, whereas some competing methods exhibit noticeable mixing across clusters in the embedding space. These observations suggest that AFTV‐LRR can effectively suppress noise and enhance the intrinsic cluster structure, thereby improving clustering performance. Although deep learning methods such as scVI and scGNN are widely used for learning nonlinear embeddings of scRNA‐seq data, and recent frameworks such as scMoMtF further extend representation learning to single‐cell multi‐omics analysis, they are not included in our main comparisons because they involve either a different training‐based pipeline or a broader task setting than single‐modality scRNA‐seq clustering. To ensure a fair and reproducible evaluation under a unified setting, we focus on representative similarity/subspace clustering baselines, and benchmarking against these broader modern approaches under a standardised and fair protocol will be an important next step for positioning AFTV‐LRR relative to modern single‐cell analysis methods.

#### 3.4.2 Computational Time Comparison

**FIGURE 6:**
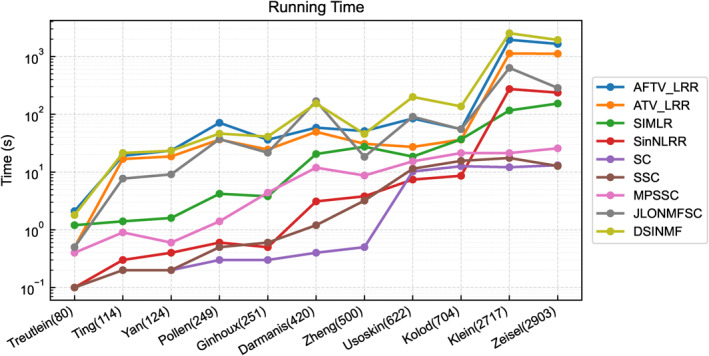
Running time comparison across 11 scRNA‐seq datasets. The *x*‐axis lists datasets with the number of cells in parentheses, and the *y*‐axis denotes wall‐clock time (seconds) in logarithmic scale. All methods were executed under the same hardware and experimental settings.

To quantify the practical applicability of the proposed method, we further report the computational time of AFTV‐LRR and the compared methods on all 11 datasets. Figure 6 shows the wall‐clock running time (in seconds) under the optimal parameter setting of each method, with the *y*‐axis in logarithmic scale due to the wide range of runtimes. All experiments were conducted on the same machine, and the reported time corresponds to the end‐to‐end clustering procedure from the preprocessed expression matrix to the final cluster assignment. For fair comparison, we set the number of iterations to 100 for each method, consistent with the experimental setting in Section 3.4.1.

As can be observed from Figure 6, the proposed AFTV‐LRR generally requires more computational time than most baselines, and the gap becomes more apparent on large‐scale datasets (e.g., Klein and Zeisel). This is expected since AFTV‐LRR incorporates the fractional‐order total variation regularisation and is solved via an iterative optimisation procedure, which increases the per‐iteration computational burden. In contrast, lightweight clustering pipelines such as SC/SSC/MPSSC typically complete within a much shorter time. Overall, these results quantify the time–accuracy trade‐off: AFTV‐LRR achieves consistently stronger clustering performance (Tables 3 and 4) at the cost of increased runtime, particularly on datasets with thousands of cells.

#### 3.4.3 Marker Gene Identification

After clustering, identifying cell‐type marker genes is essential for accurate cell‐type annotation in scRNA‐seq analysis. Marker genes are typically employed to characterise predefined or novel cell types, and their identification largely depends on the reliability of clustering outcomes.

**FIGURE 7:**
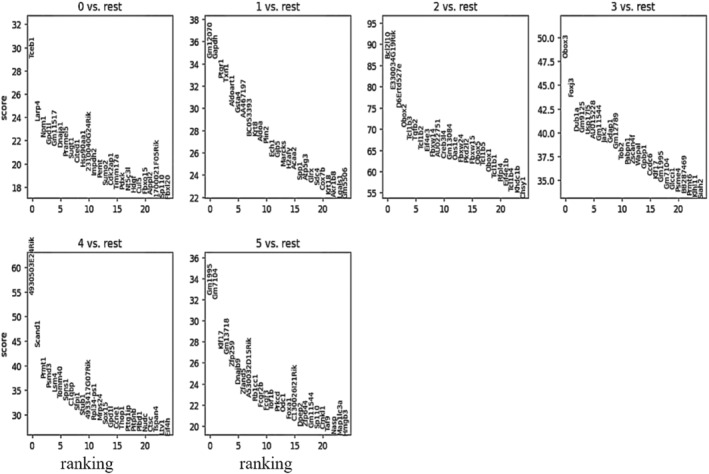
Top 25 ranked marker genes for each cell cluster based on *t*‐test scores. The x‐axis denotes gene ranking, and the y‐axis denotes the corresponding test score.

In this study, the *mouse embryo* dataset [46], which contains 268 cells and 22,431 genes across six distinct cell types, was utilised for marker gene identification. A two‐sample *t*‐test was applied to compute the differential expression score of each gene across clusters, ranking genes based on statistical significance. The top 25 genes with the highest scores were selected as marker gene candidates for each cluster. As shown in Figure 7, the horizontal axis represents the ranked marker genes, and the vertical axis indicates the corresponding *t*‐test scores for each cluster.

To further investigate the expression patterns of these marker genes, two representative genes among the top 25 for each cluster were selected and visualised using both bubble plots and violin plots. The bubble plots illustrate the proportion of expressing cells (bubble size) and the average expression level (colour intensity), whereas the violin plots show the distribution of expression values across clusters. Combining these two visualisation methods highlights both the overall abundance and the heterogeneity of marker gene expression among clusters.

**FIGURE 8:**
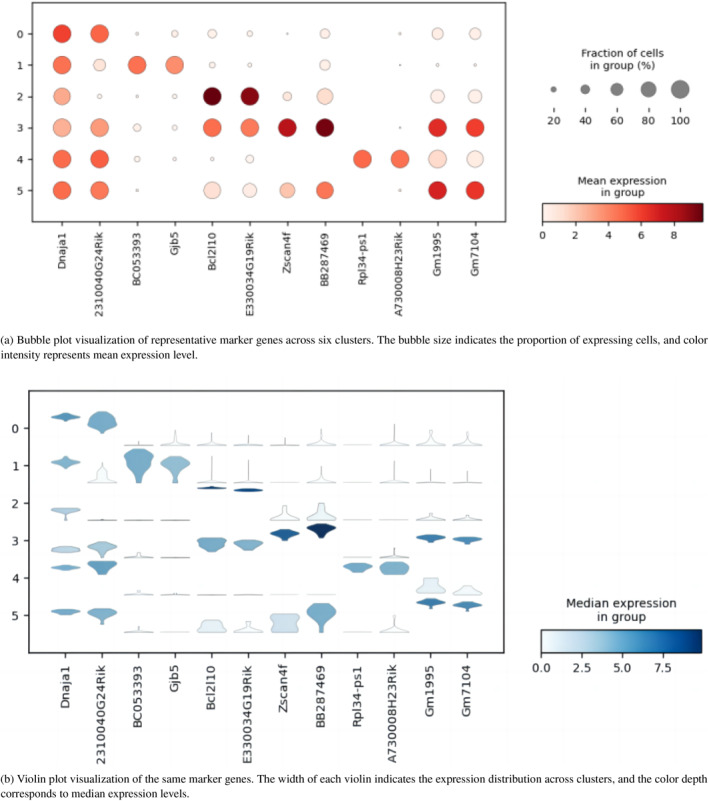
Visualisation of marker gene expression patterns across six cell clusters. (a) Bubble plots and (b) violin plots together demonstrate clear cluster‐specific expression signatures of marker genes.

From Figures 7 and 8, the identified marker genes exhibit clear cluster‐enriched expression patterns, suggesting that the obtained clusters are biologically interpretable. Moreover, functional enrichment analysis further supports biological coherence of the clusters by revealing cluster‐specific GO biological processes. Together, these results indicate that AFTV‐LRR yields clustering outcomes that are not only statistically supported but also biologically meaningful for downstream cell‐type annotation.

#### 3.4.4 Functional Enrichment Analysis

To provide biological validation beyond differential‐expression statistics, we performed functional enrichment analysis for each cluster using g:Profiler (g:GOSt). For each cluster, the top 25 marker genes were used as input, and enriched Gene Ontology Biological Process (GO:BP) terms were identified with Benjamini–Hochberg correction (BH‐FDR <0.05). Overall, the enriched terms revealed coherent and cluster‐specific functional programs, supporting the biological interpretability of the identified cell groups. Representative terms for each cluster are summarised in Table 5.

**TABLE 5:** Representative GO:BP enrichment terms for each cluster identified by g:Profiler (g:GOSt) with Benjamini–Hochberg correction (BH‐FDR <0.05).

Cluster	Representative term	GO ID	BH‐FDR
0	Nucleocytoplasmic transport	GO:0006913	$6.88\times 10^{-5}$
1	Cell‐substrate adhesion	GO:0031589	$1.69\times 10^{-4}$
2	Maternal‐to‐zygotic transition of gene expression	GO:0160021	$1.59\times 10^{-3}$
3	Regulation of DNA‐templated transcription	GO:0006355	$5.68\times 10^{-5}$
4	Regulation of chaperone‐mediated protein complex assembly	GO:0090034	$4.21\times 10^{-3}$
5	Cellular response to reactive oxygen species	GO:0034614	$1.12\times 10^{-3}$

As shown in Table 5, Cluster 0 was enriched in nucleocytoplasmic transport, Cluster 1 was associated with cell–substrate adhesion, Cluster 2 was linked to maternal‐to‐zygotic transition of gene expression, Cluster 3 highlighted transcriptional regulation, Cluster 4 suggested chaperone‐mediated proteostasis programs, and Cluster 5 was related to oxidative‐stress response. These results provide complementary biological evidence for functional heterogeneity among the identified clusters.

## 4 Conclusions and Future Work

In this study, we proposed an Adaptive Fractional‐Order Total Variation Regularised Low‐Rank Representation (AFTV‐LRR) model for clustering single‐cell RNA sequencing (scRNA‐seq) data. By incorporating adaptive fractional‐order total variation into the low‐rank representation framework, AFTV‐LRR enables reliable recovery of intrinsic subspace structures while preserving local geometric characteristics. Through fractional‐order gradient learning, the proposed method provides strong denoising capability and retains biologically meaningful signals. The resulting optimisation problem is efficiently solved via the Alternating Direction Method of Multipliers (ADMM), and spectral clustering is subsequently applied to identify distinct cell populations.

Extensive experiments on 11 publicly available scRNA‐seq datasets demonstrate that AFTV‐LRR achieves *competitive and often superior* performance compared with eight representative clustering algorithms, including ATV‐LRR, SIMLR, SinNLRR, JLONMFSC, and DSINMF, in terms of Adjusted Rand Index (ARI) and Normalised Mutual Information (NMI). Visualisation results further indicate that AFTV‐LRR generally yields clearer inter‐cluster separation and higher intra‐cluster compactness. Moreover, marker genes identified from the inferred clusters exhibit strong biological specificity, supporting the interpretability and robustness of the proposed framework. Overall, these results highlight the potential of AFTV‐LRR for cell type discovery and for facilitating downstream biological investigations.

Despite its favourable performance, AFTV‐LRR has several limitations. The introduction of fractional‐order regularisation increases computational complexity, resulting in longer runtimes on large‐scale datasets. In addition, the selection of key hyperparameters—particularly the fractional order and neighbourhood size—still relies on empirical tuning, which may limit full automation across diverse datasets and experimental conditions.

Future work will focus on improving computational efficiency through accelerated optimisation strategies and GPU‐based parallelisation. We also plan to extend AFTV‐LRR to multi‐omics integration and spatial transcriptomics, where adaptive fractional‐order modelling may better capture spatial organisation and regulatory dependencies among cells. These directions are motivated by recent advances in interpretable single‐cell multi‐omics learning and unsupervised spatial transcriptomics analysis [25, 26]. Furthermore, we will investigate combining AFTV‐LRR with deep representation learning or graph neural network priors to better model complex nonlinear cellular relationships. In particular, benchmarking AFTV‐LRR against modern deep learning‐based methods (e.g., scVI, scGNN, and related representation‐learning frameworks) under a standardised evaluation protocol with controlled computational settings will be an important next step for clearly positioning the proposed method relative to current state‐of‐the‐art approaches. We will also explore self‐supervised pretraining strategies and transformer‐based single‐cell language modelling to further enhance generalisation on large‐scale and noisy datasets [30]. In addition, recent progress in single‐cell chromatin accessibility analysis and dynamic multi‐view clustering suggests that more expressive representation learning and fusion mechanisms may further improve the scalability and adaptability of AFTV‐LRR in broader omics settings [27, 28, 29]. These developments are expected to make AFTV‐LRR more scalable, interpretable, and broadly applicable for comprehensive single‐cell data analysis and biological discovery.

## Author Contributions

**Pengcheng Yang:** conceptualization, data curation, formal analysis, methodology, project administration, software, validation, visualization, writing – original draft. **Fei Lu:** formal analysis, methodology, validation, writing – original draft. **Qianwen Xue:** formal analysis, methodology, validation. **Weimin Ma:** conceptualization, data curation, writing – original draft. **Qianwen Liu:** formal analysis, methodology, validation, writing – original draft. **Qiang Li:** formal analysis, methodology, validation, writing – original draft. **Yulin Zhang:** conceptualization, data curation, formal analysis, writing – original draft. **Xiaochun Cheng:** formal analysis, investigation, software, supervision, validation, writing – original draft.

## Funding

Authors were supported by UKRI Grant EP/W020408/1.

## Ethics Statement

All single‐cell RNA sequencing datasets used in this study are publicly available and were obtained from previously published studies. No new human or animal experiments were conducted by the authors. Ethical approval and informed consent for each dataset were obtained by the original investigators as described in their respective publications.

## Conflicts of Interest

The authors declare no conflicts of interest.

## Code Availability

The implementation of AFTV‐LRR is publicly available at https://github.com/yangpengcheng7/AFTV‐LRR.

## Data Availability

Data will be made available on request.
